# Risk Factors for Immune Checkpoint Inhibitor–Mediated Cardiovascular Toxicities

**DOI:** 10.1007/s11912-023-01414-4

**Published:** 2023-04-20

**Authors:** Laura I. Yousif, Elles M. Screever, Daniëlle Versluis, Joseph Pierre Aboumsallem, Stefan Nierkens, Olivier C. Manintveld, Rudolf A. de Boer, Wouter C. Meijers

**Affiliations:** 1 Department of Cardiology, Thorax Center, Erasmus University Medical Center, P.O. Box 2040, 3000CA Rotterdam, The Netherlands; 2 Graduate School of Life Science, Utrecht University, P.O. Box 80125, 3508 TC Utrecht, The Netherlands; 3 Center for Translational Immunology, Utrecht University, University Medical Center Utrecht, P.O. Box 85500, 3508 GA Utrecht, The Netherlands; 4 Princess Máxima Center for Pediatric Oncology, Heidelberglaan 25, 3584CS Utrecht, The Netherlands

**Keywords:** ICI, Myocarditis, Pericarditis, Vasculitis, Risk factors

## Abstract

**Purpose of Review:**

Immune checkpoint inhibitors (ICIs) have improved the field of cancer, especially in patients with advanced malignancies. Nevertheless, cardiovascular immune-related adverse events (irAEs) with high mortality and morbidity have been observed, including myocarditis, pericarditis, and vasculitis. To date, only a few clinical risk factors have been described and are currently being investigated.

**Recent Findings:**

In this review, we address the four most prevailing risk factors for cardiovascular irAEs. ICI combination therapy is a predominant risk factor for developing ICI-mediated myocarditis. Additionally, ICI combined with other anti-cancer treatments (e.g., tyrosine kinase inhibitors, radiation, chemotherapy) seems to increase the risk of developing cardiovascular irAEs. Other risk factors include female sex, pre-existing cardiovascular disease, and specific tumors, on which we will further elaborate in this review.

**Summary:**

An a priori risk strategy to determine who is at risk to develop these cardiovascular irAEs is needed. Insights into the impact of risk factors are therefore warranted to help clinicians improve care and disease management in these patients.

## Introduction

**Fig. 1:**
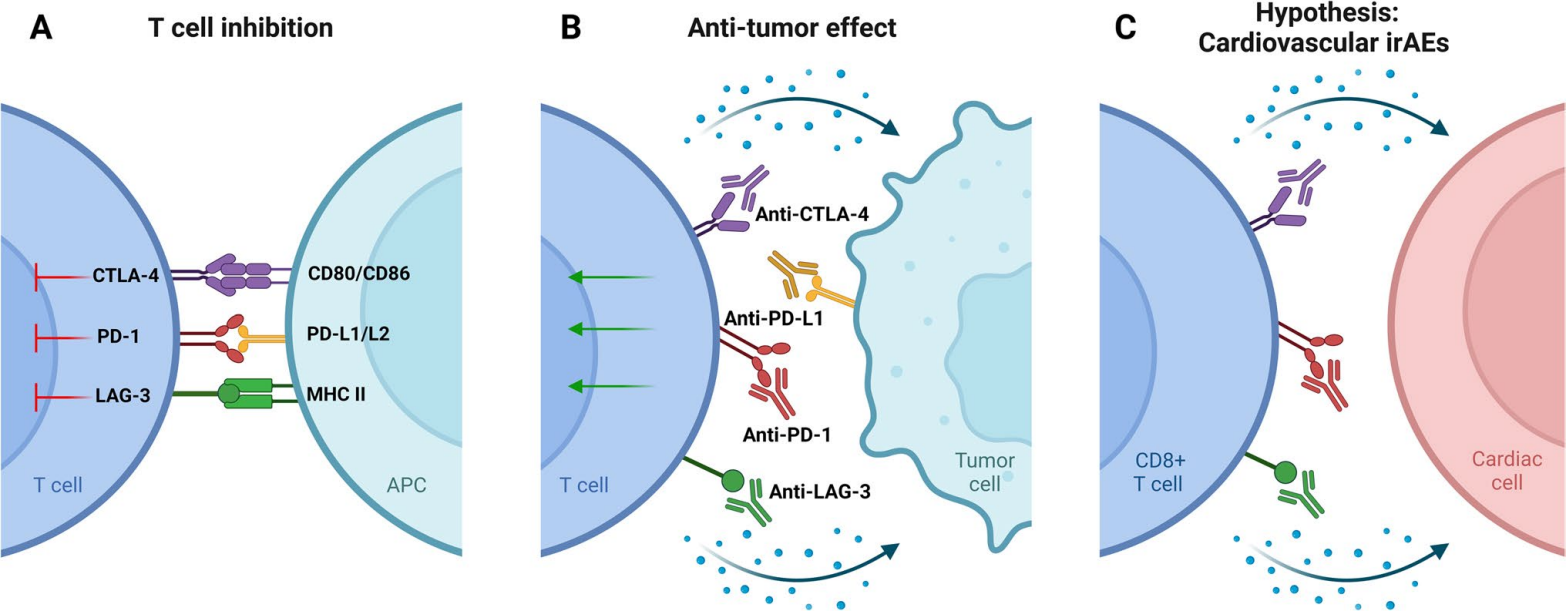
ICI mechanisms of action. **A** CTLA-4, PD-1, and LAG-3 binding leads to T cell inhibition. **B** Immune checkpoint inhibitors (ICI) block T cell inhibition, allowing for T cell activation in the tumor microenvironment (incl. cytokine release) and a potent anti-tumor response. **C** It is hypothesized that cardiovascular immune-related adverse events (irAEs) could occur through direct interaction between T cells and cardiac cells. Created with Biorender. CTLA-4, Cytotoxic T-lymphocyte-associated protein 4; PD-1, programmed cell death protein-1; PD-L1/L2, programmed cell death protein-1-ligand 1/2; LAG-3, lymphocyte activation gene-3; MHC-II, major histocompatibility complex II

Immune checkpoint inhibitors (ICIs) have revolutionized cancer treatment of a variety of malignancies during the past decades. In physiological conditions, immune checkpoints (ICs), cytotoxic T-lymphocyte associated protein 4 (CTLA-4), programmed cell death protein 1 (PD-1) and its ligand PD-L1, and lymphocyte activation gene-3 (LAG-3) on the T cell surface act as gatekeepers of T cell activation or inhibition. Once bound to their corresponding ligands on other cells, they inhibit T cell activation and the subsequent immune response (Fig. 1A) [1]. Without the presence of these ICs, an excessive immune response could lead to autoimmunity; therefore, they safeguard self-tolerance. As tumor cells and cells in the tumor microenvironment (TME) can express IC ligands in order to avoid immune surveillance and an effective T cell–mediated anti-tumor response, ICIs were developed to block inhibition of ICs and thwart this ability (Fig. 1B) [1]. ICI therapy is known to significantly improve clinical outcome and survival rates in patients with advanced stage tumors [2, 3]. To date, the US Food and Drug Administration (FDA) has approved 8 ICIs, on the abovementioned ICs, for the treatment of numerous tumor types as a first-line treatment as well as an adjuvant therapy.

Although ICIs are a great success with regard to prognosis, there is a substantial risk of developing immune-related adverse events (irAEs) [4]. The effects of ICIs are not confined to the tumor and TME but are systemic and can have a wide range of adverse effects on organs, ranging in severity from asymptomatic to life-threatening. Cardiovascular irAEs (Fig. 1C), including myocarditis, pericarditis, and vasculitis, currently occur in approximately ~ 1–1.5% of the patients and, while infrequent, the mortality rate is up to 50% in the case of myocarditis [5, 6]. For pericarditis and vasculitis, mortality rates are approximately 21% and 6%, respectively. Macrophage and T cell infiltration characterize myocarditis and pericarditis, but also the acceleration of atherosclerosis due to vasculitis [7–9]. In the potential ramifications on disease progression (i.e., acceleration of atherosclerosis), these cardiovascular irAEs can therefore also have long-term effects on the lives of these patients.

Unravelling the underlying mechanisms causing these life-threatening cardiac irAEs could give new insights to identify those people who are susceptible. In this review, we summarize the most important risk factors for ICI-mediated myocarditis, pericarditis, and vasculitis.

## Treatment Regimen

### ICI Monotherapy vs Combination Therapy

A predominant risk factor observed for cardiovascular irAEs is the treatment regimen. Despite tremendous advances of ICI monotherapy in patients with advanced malignancies, the patient response rate only ranges from 20 to 40% [10, 11]. This resistance to ICI treatment can be either primary or secondary. Primary resistance implies that the tumor is resistant to the ICIs used. With secondary resistance, the drugs elicit an initial short-lived anti-tumor effect before the tumor adjusts to circumvent immune activation otherwise and renders the ICI ineffective [12]. Combining ICIs has proven effective against these resistant tumors, though in certain cases, this is accompanied by an increased risk of cardiovascular irAEs. In general, an increased incidence of developing cardiovascular irAEs was seen with combination therapy, 5.8% [3.86–9.01], compared to ICI monotherapy, 3.1% [0.73–7.06], highlighting myocarditis as the most lethal manifestation [13•].

**Fig. 2:**
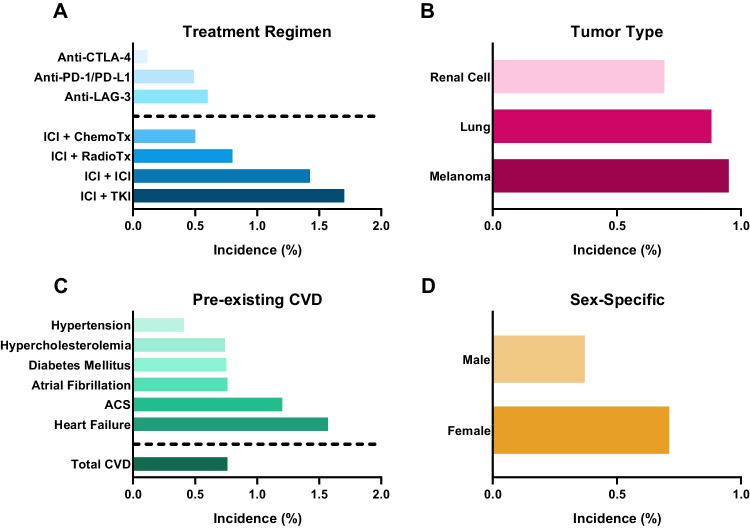
A schematic overview of risk factors for ICI-mediated myocarditis. **A** ICI in combination with ICI or other cancer treatment is associated with higher incidence of ICI-mediated myocarditis. **B** Melanoma is the most common cancer in patients with ICI-mediated myocarditis. **C** Pre-existing cardiovascular disease (CVD) has been reported in the majority of people with ICI-mediated myocarditis, with most incidence in patients with heart failure and acute coronary syndrome (ACS). **D** ICI-mediated myocarditis has a higher incidence in females. CTLA-4, cytotoxic T-lymphocyte-associated protein 4; PD-1, programmed cell death protein-1; PD-L1, programmed cell death protein-1-ligand 1; LAG-3, lymphocyte activation gene-3; TIK, tyrosine kinase inhibitor; ChemoTx, chemotherapy; RadioTx, radiotherapy. Data derived from [5, 6, 17••, 34, 39–41, 61]

Given the highly fatal consequences of fulminant myocarditis following ICI treatment, it is the most extensively studied cardiovascular irAE. A pharmacovigilance study using the World Health Organization’s (WHO) global database of case safety reports in 2018 found that myocarditis occurred in 0.41% of all case safety reports for anti-PD-1/PD-L1 (e.g., nivolumab and atezolizumab) compared to 0.07% for anti-CTLA-4 (e.g., ipilimumab) with a reporting odds ratio (ROR) of 5.62 [2.46–12.88] [5]. In accordance, another study using the updated WHO database in 2021 found that anti-PD-1 monotherapy specifically has been found to induce most of the ICI-mediated myocarditis, with an incidence of 0.57% for anti-PD-1 compared to 0.16% for anti-CTLA-4 [13•]. Interestingly, already in 2016, two cases of severe myocarditis with fatal outcome in melanoma patients treated with ipilimumab and nivolumab combination treatment were reported [14]. ICI combination therapy (at least one anti-PD-1/PD-L1 with anti-CTLA-4) was associated with higher reporting of myocarditis than ICI monotherapy with a ROR of 4.31 [2.86–6.38] [5]. Myocarditis incidence rates were then determined for patients on nivolumab or nivolumab/ipilimumab treatment using the Bristol-Myers Squibb corporate safety database, showing incidence rates of 0.06% and 0.27% (*p* < 0.001), respectively. Additionally, while 10% (*n* = 10) of cases were fatal in the nivolumab mono-treatment group, 63% (*n* = 8) died from myocarditis in the nivolumab/ipilimumab group [14]. In another study, myocarditis was more common in the ICI combination treatment group compared to anti-PD-1 monotherapy, 2.4% and 0.5%, respectively [6] 6. Figure 2A provides a schematic overview of myocarditis incidences based on treatment regimens.

The first LAG-3 ICI, relatlimab, was FDA-approved at the beginning of 2022 and therefore data regarding combination treatment is still limited. In a murine study, LAG-3 knockout mice did not develop autoimmune disease, while their LAG-3/PD-1 double knockout counter group developed severe myocarditis with myocardial T cell infiltration, underlining the highly likely cardiotoxic ramifications of ICIs [15, 16]. Additionally, mouse tumor analysis revealed high co-expression of LAG-3 and PD-1 on tumor-infiltrating T cells, hereby highlighting LAG-3 as a potential candidate to overcome anti-PD-1/PD-L1 resistance. Indeed, concurrent nivolumab and relatlimab treatment increased progression-free survival as compared to nivolumab alone in a randomized phase 3 clinical trial [17••]. However, myocarditis incidence rates were almost threefold in nivolumab/relatlimab patients in comparison with the nivolumab group, which is comparable to what is observed for combined PD-1/PD-L1 and CTLA-4 inhibition. It is important to note that while anti-LAG-3 appears to increase the incidence of ICI-mediated myocarditis in the schematic overview of treatment regimen incidence (Fig. 2A), this observed effect is still only from limited data. Furthermore, as represented in Fig. 2A, inhibition through of the combination therapy appears to be the predominant drug risk factor in ICI-mediated myocarditis.

Pericardial disease, including pericarditis and pericardial effusion, can lead to life-threatening cardiac tamponade. In a retrospective single-center study, an incidence rate of 1.57% has been reported for pericardial disease with threefold increase in pericarditis incidence in ICI-treated patients compared to non-ICI-treated patients [18]. This study showed a more than fourfold increased risk for development of pericarditis and pericardial effusion, even when adjusted for potential confounders (hazard ratio HR) 4.37 [2.09–9.14, *p* < 0.001]. Additionally, ICI-mediated pericardial disease was associated with a 1.5-fold mortality risk. In a recent systematic review of case studies, anti-PD-1 therapy was used in all patients that presented with ICI-mediated pericarditis [19]. As with myocarditis, pericarditis due to anti-PD-1 or anti-PD-L1 monotherapy occurred more frequently than with anti-CTLA-4 (0.36% and 0.16%, respectively) with a ROR of 2.28 [1.27–4.12] [5].

ICI treatment was found to be associated with higher reporting of vasculitis, ROR 1.56 [1.25–1.94] [5]. A case study linked myocardial injury to myocardial vasculitis with the use of pembrolizumab (anti-PD-1) [20]. Researchers observed enhanced T cell infiltration and production of proinflammatory cytokines in the hearts of LAG-3/PD-1 deficient mice, suggesting that inhibition of both ICs in patients presumably will be associated with enhanced risk of developing myocarditis, pericarditis, and vasculitis [16]. Contrarily, cardiotoxicity risk due to ICI combination therapy was not found with neither pericarditis (or pericardial disease in general) nor vasculitis, with RORs of 1.1 [0.53–2.24] and 1.3 [0.62–2.67], respectively [18, 21, 22]. While this could be due to underreporting of these irAEs, it also suggests that cardiovascular irAEs can be specific to certain affected ICs.

Currently, new promising ICIs are under investigation in several clinical and preclinical studies [12]. Among them are antibodies against T cell immunoglobulin and ITIM domain (TIGIT) and T cell immunoglobulin and mucin-domain containing-3 (TIM-3) as the next-generation targets [23]. Preclinical models already showed promising results regarding anti-tumor response with TIM-3 and PD-1/PD-L1 combination treatment [24]. Future research needs to be performed to see whether these ICIs are as effective as, and less cardiotoxic than, current ICIs.

#### ICI in Combination with Other Cancer Therapies

Clinical indications towards ICI are expanding rapidly and accumulating evidence suggests synergetic anti-tumor effect of ICI treatment combined with other treatment modalities. These combinations, whether concomitant or sequential, have been authorized for a range of tumors to improve immunotherapy effectiveness. Prolonged progression-free survival was reported in advanced non-small cell lung cancer (NSCLC) patients treated with anti-PD-1 with concomitant chemotherapy or radiation therapy compared to ICI alone [25–28]. However, these conventional cancer treatment modalities are known to have their own cardiotoxicity and concomitant treatment with ICI therapy might result in cumulative cardiotoxicity.

Radiation therapy causes DNA damage and the production of free radicals, resulting in immunogenic cancer cell death. Additionally, it gives a boost to immunity by inducing MHC I upregulation and cytokine release [29]. The synergistic anti-tumor response from concomitant radiation and ICIs works through immunogenic cell death in combination with removal of the immune cell breaks and elicits a stronger immune response. Consequently, however, this could also affect off-target cells, causing (cardiovascular) irAEs. In a mouse model, concomitant cardiac radiation and anti-PD-1 treatment showed increased cardiotoxicity compared to cardiac radiation alone. Cardiotoxicity translated into acute mortality of 30% and was associated with reduced cardiac output and increased CD8 + T cell infiltration and fibrosis [29]. The observed reduced cardiac output and, especially, myocardial CD8 + T cell infiltration mimic the clinical setting. In another experimental study, C57Bl/6 mice were treated with thoracic radiation and either a control IgG or anti-PD-1. Mice in the anti-PD-1/radiation group showed reduced survival and elevated T cell infiltrates in both the heart and lungs [30]. This observation is supported by a phase 1 trial in which patients who received any thoracic radiation therapy prior to ICI were more likely to develop any grade pulmonary toxicity, including pneumonitis [27]. While this study only reported on pulmonary toxicities, it provides evidence to speculate that thoracic radiation therapy with cardiac involvement increases the risk of developing ICI-mediated myocarditis and pericarditis.

Chemotherapy, especially anthracyclines, is known to be highly cardiotoxic [31]. A systematic review and meta-analysis recently found that combined ICI and chemotherapy treatment significantly increased the incidence of cardiovascular irAEs in general with a pooled risk ratio of 1.68 [1.07–2.64, *p* = 0.026] [32]. In another meta-analysis, the incidence of cardiovascular irAEs in general was higher for patients receiving concomitant ICI and chemotherapy as compared to solely ICI or chemotherapy, with incidences of 3.7%, 3.1%, and 2.5%, respectively [13•]. Contradictory results were reported in a retrospective study where no significant difference has been found for myocarditis and pericarditis [33]. A possible explanation for this could be different compositions of treatment regimen between cohorts with regard to specific kinds of ICIs or chemotherapy [34].

Another class of cancer treatment is tyrosine kinase inhibitors (TKI), including vascular endothelial growth factor (VEGF) inhibitors such as sunitinib and axitinib, which are standard first-line therapy for renal cell carcinoma and have been associated with heart failure [35]. A single-group, phase 1b trial compared concomitant axitinib (VEGF inhibitor) and avelumab (anti-PD-L1) with only sunitinib treatment in patients with renal cell carcinoma [36]. Concomitant axitinib and avelumab treatment nearly doubled the median progression-free survival, 13.8 months for axitinib/avelumab compared to 7.2 months for sunitinib, with a striking increase in the objective response rate in patients with PD-L1 positive tumors, 55.2% and 22.5% respectively. Interestingly, more irAEs occurred and lead to discontinuation of treatment in the sunitinib group as compared to the axitinib/avelumab group, 13.4% and 7.6% respectively. Here, once again, death due to treatment toxicity in the combination group included death attributable to myocarditis. Additionally, a meta-analysis found significant associations with myocarditis in patients treated with axitinib and both pembrolizumab (ROR 36.9 [11.8–115.9]) as well as avelumab (55.6 [13.4–222.3]) [34]. Possible explanations behind the potentially detrimental cardiotoxic synergy here are that VEGF inhibitors are known to cause hypertension and are associated with coronary ischemia and thrombosis [37]. Given the numerous clinical trials on the combination of TKI and ICI treatments, and the presumable toxic effect on vasculature, cardiovascular irAEs should be monitored closely [38].

All in all, these findings illustrate the crucial role that proper treatment can play in the development of cardiovascular irAEs following ICI treatment, whether alone, in combination with other ICI or other cancer therapies.

## Tumor Type

The likelihood of developing specific cardiovascular irAEs might differ depending on the specific tumor type. Cardiovascular irAEs are frequently reported among patients with melanoma or lung cancer, with a retrospective study that examined differences in cardiovascular irAEs across different cancer types observing that patients who developed irAEs within 1 year of ICI initiation were more likely to have lung cancer than melanoma (42.3% vs 34.7%. *p* < 0.0001) [39]. More into detail, comparison of event rates between lung malignancy types revealed that more cases of cardiac irAEs have been reported for patients with NSCLC (11.4%) compared to small cell lung cancer patients (6,2%) and only a few reported cases in patients with other malignancies [40]. However, there appears to be variation among prospective cardiovascular irAEs. Pericardial disease was most reported in lung cancer patients (56%) with a hazard ratio of 5.46 [2.96 to 10.10] [5]. On the other hand, myocarditis and vasculitis were more reported in patients with melanoma, 41% and 60%, respectively (Fig. 2B). This observation that myocarditis and vasculitis are more prevalent in patients with melanoma, and pericarditis in patients with lung cancer was also observed more often [41]. While ICIs are more often administered as treatment for melanoma and lung cancer, presumably causing over reporting of these specific malignancies, the underlying mechanism causing this variation remains to be elucidated. Another reasonable explanation for the increased incidence of pericarditis in lung cancer patients is because it is a common side effect of lung cancer regardless of treatment [42].

Different irAE profiles have been associated with specific cancer types, including melanoma, renal cell carcinoma, and lung cancer, when treated with anti-PD-1 [43]. Melanoma patients showed higher frequency of skin and gastrointestinal irAEs while lower frequency of pneumonitis compared to NSCLC. A clear explanation for this observation remains elusive; however, the TME, neoantigen formation, and immune cell infiltration may be influenced by specific tumor types with different toxicity profiles as a consequence. Unfortunately, cardiovascular irAEs were not assessed in this study [43]. Tumors with high tumor mutation burden (TMB), including melanoma and NSCLC, are associated with higher risk of irAEs compared to low TMB malignancies [44]. Antigenicity will increase due to this high TMB, resulting in an increased probability of T cell cross-reaction with healthy tissue antigens. Interestingly, high TMB malignancies are also associated with increased responsiveness to ICI treatment. Therefore, experiencing irAEs might be double-edged since it is also associated with better progression-free survival, even among patients with metastatic cancer [45, 46]. For example, patients with metastatic renal cell carcinoma experiencing irAEs of any kind or severity had better response rates to ICIs as well as significantly longer progression-free survival (13.1 vs. 4.87 months, *p* = 0.0001) and overall survival (overall survival: 26.0 vs. not reached, *p* = 0.0072) than patients without irAEs [47]. Thus, tumor type seems to be correlated with specific cardiovascular irAEs and experiencing irAEs could be used as a predictive factor for patient outcome when treated with ICI.

### Self-Antigens

It is hypothesized that cancer cells could produce and secrete antigens that are also expressed by cardiac or vascular tissue, thus increasing the chance of an immune cross-reaction in the cardiovascular organs. There is growing evidence that extensive T cell clonal expansion causes the appearance of self-reactive T cells, resulting in cardiovascular toxicities [48]. Indeed, in a transgenic *Ctla4*^+*/−*^*Pdcd1*^*−/−*^ mouse model which resembles ICI-mediated myocarditis in patients, T cell receptor (TCR) sequencing revealed an accumulation of T cells with α-myosin-reactive TCRs in the hearts of these mice [49, 50•]. This implies that α-myosin heavy chain is an important self-antigen in ICI-mediated myocarditis in mice. A second mouse model which induced myocarditis with solely anti-PD-1 inhibition also observed accumulation of α-myosin-reactive T cells in the hearts, further supporting the role of self-antigens in ICI-mediated myocarditis [51]. To translate this to humans, the presence of these α-myosin-reactive TCRs has been confirmed in patients and controls. Specific α-myosin-reactive TCRs were present in the inflamed heart and skeletal muscle, indicating a relevant disease antigen for ICI-mediated myocarditis and myositis. Analysis of an already published RNA-seq database revealed that tumors could also express low but detectable levels of *MYH6*, the gene expressing α-myosin heavy chain, in melanoma [50•, 52]. Whether tumor expression of *MYH6* is a risk factor for developing ICI-mediated myocarditis or myositis remains to be elucidated due to the relatively small patient cohort in this study. Interestingly, diabetic mice with a myocardial infarction had increased myocardial infiltration of T cells directly responsive against α-myosin heavy chain [53]. This supports the notion of myocardial injury-mediated antigen exposure with ensuing T cell–mediated damage. These findings also provide interesting perspectives on the potential role of self-antigens as biomarkers for determining patient susceptibility to cardiotoxicity.

## Pre-existing Cardiovascular Disease

Pre-existing cardiovascular disease (CVD) in cancer patients treated with ICI is another interesting risk factor. In a meta-analysis, a substantial fraction of all the cases (40%) that experienced cardiovascular irAEs presented cardiovascular risk factors [13•]. Among them, myocardial infarction, peripheral coronary artery disease, and hypertension were the most frequent reported manifestations. In a retrospective study with three ICI-mediated myocarditis cases, two out of three patients experienced previous CVD, including myocardial infarction, aortic aneurysm, and hypertension [54]. Comparable observations were found in a case series study where pre-existing CVD was more prevalent in the patient group (63%) [55]. Moreover, two fatal cases of myocarditis were reported and both patients had a history of hypertension, although without other cardiovascular risk factors [7]. A Cox proportional-hazard model showed that history of acute coronary syndrome (ACS) (HR 4.06, 95% CI 1.15 to 14.3* p* = 0.03) and heart failure (HR 5.2, 95% CI 1.4 to 18.7, *p* = 0.01) was associated with a higher incidence of myocarditis (Fig. 2D) [41]. A risk prediction model was formulated with these risk factors and older age, showing a cumulative risk of ICI-mediated myocarditis. In this study, diabetes mellitus (DM) and hypertension were also common in the myocarditis group, 58% and 42%, respectively. However, no direct comparison was made between the ICI-treated and non-ICI-treated groups [41].

This accumulating evidence suggests pre-existing CVD as a potential risk factor for developing cardiovascular irAEs, especially ICI-mediated myocarditis.

Another potential risk factor that has been studied is DM. While two independent studies were published in which no significant association between DM and ICI-mediated myocarditis was found, there is plenty of research that suggests otherwise [56, 57]. DM was reported to be more common in ICI-mediated myocarditis cases compared to controls, 34% and 13%, respectively (*p* = 0.01). Another multivariate regression analysis suggested that DM (OR = 1.96, 95% CI: 1.05–3.65, *p* = 0.034) was an independent risk factor for the occurrence of ICI-related cardiotoxicity [58]. As previously mentioned, also with regard to DM, research has indicated a potential role for self-antigens, specifically α-myosin heavy chain, in the development of cardiovascular irAEs [53]. Considering the various interfaces between CVD and DM, and the three irAEs discussed in this review, evidence suggesting that diabetic patients might be at a higher risk should be taken into account and further investigated.

## Sex Specificity

In general, males are known to be at higher risk of developing viral or non-ICI-mediated myocarditis, whereas females are more likely to develop autoimmune disease in general, suggesting a predisposition to ICI-mediated myocarditis or cardiovascular irAEs [59]. Indeed, female sex has an increased incidence ratio of developing cardiovascular irAEs of 3.34 [1.42–7.85, *p* = 0.006). These results were supported by another study where females experienced more cardiovascular irAEs in general (*p* = 0.011), including pericarditis [57]. Vasculitis was found to not be associated with sex, although this is limited to data from a single systematic review [60]. With regard to myocarditis, in a transgenic *Ctla4*^+*/*^*Pdcd1*^*−/−*^ mouse model mimicking ICI-mediated myocarditis, greater mortality and a more severe disease course were observed in female mice [49]. In humans, an observational study using the FEARs database reported that besides an age older than 75 (OR, 7.61; 95% CI, 4.29–13.50; *p* < 0.001), female sex (OR, 1.92; 95% CI, 1.24–2.97; *p* = 0.004) was also significantly associated with ICI-mediated myocarditis (Fig. 2C) [61].

A recently published study investigated the underlying mechanism behind these sex-specific differences in mice [62]. Female tumor-bearing mice treated with a combination of anti-PD-L1 and anti-CTLA-4 antibodies demonstrated more pronounced myocardial CD8 + lymphocyte infiltration than male ones. In-depth analysis of the hearts showed decreased expression of mesencephalic astrocyte–derived neurotrophic factor (Manf) and heat shock 70 kDa protein 5 (Hspa5) in ICI-treated mice compared to non-ICI-treated mice, which was confirmed using the *Ctla4*+/−*Pdcd1*−/− mouse model [49]. Furthermore, decreased expression of those proteins was also found in human heart tissue. Notably, more pronounced downregulation of Manf in females was a direct result of reduced β-estradiol levels following ICI treatment. The regulatory promotor region of Manf contains androgen- and estrogen-responsive elements, thus indicating a regulatory role of these hormones in the expression of Manf [62]. This indicates that female sex is a risk factor for developing ICI-mediated myocarditis and that future research is needed to elucidate this association for pericarditis and vasculitis.

### Autoimmune Disease

An interesting addition to sex-specific risk factors is autoimmune disease, given the biological predisposition of females. The main concern of providing ICI therapy to patients with underlying autoimmune disease is further aggravation of their already dysregulated immune system. While clinical trials normally provide information regarding drug safety and efficacy in patients, in most cases patients with pre-existing autoimmune disease are excluded from clinical trials and therefore safety profiles for this specific group remain unclear [7].

Several retrospective studies have examined the efficacy and safety of ICI treatment in patients with pre-existing autoimmune diseases. In one study, 50% of the patients treated with ipilimumab for advanced melanoma with baseline autoimmune disease experienced autoimmune flares or irAEs [63]. Additionally, in another study, 38% of advanced melanoma patients experienced exacerbation of their autoimmune disease, requiring immune suppression, and 29% developed irAEs [64]. In another retrospective study with NSCLS patients with autoimmune disease, 38% developed irAEs when on anti-PD-1/PD-L1 treatment, but only 5% developed both flares and irAEs [65]. Remarkably, in all three studies, despite the irAEs and flaring of symptoms, only a small fraction of the treated patients needed discontinuation of the treatment. Finally, no cardiovascular irAEs were observed in any of these studies, which might be a result of the low incidence of these irAEs and the small sample size of these abovementioned studies. Researchers have hypothesized that patients with previous autoimmune disease with cardiac involvement, including systemic lupus erythematosus, rheumatoid arthritis, sarcoidosis, and Dessler’s syndrome, might be at higher risk of developing cardiovascular irAEs [66].

## Conclusion

### Clinical Perspective

**Fig. 3:**
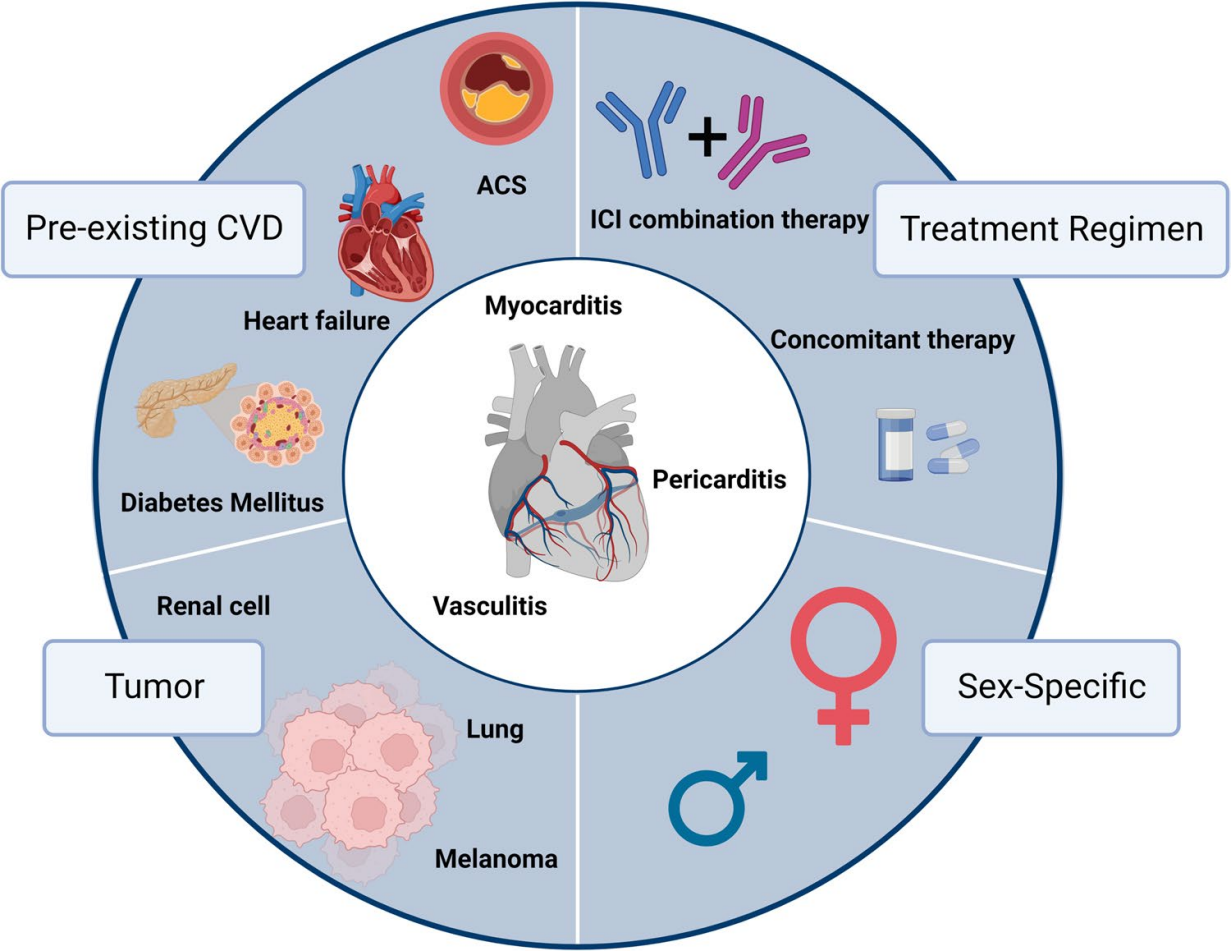
Potential risk factors for ICI-mediated myocarditis, pericarditis and vasculitis. Treatment regimen has been associated with the most cases of cardiovascular irAEs. Combination therapy seems specific for myocarditis, whereas anti-PD-1/PD-L1 mono-treatment seems most harmful in all three injuries. The female sex is implied to be a risk factor, most likely due to the autoimmune nature of cardiovascular irAEs. There also appears to be a trend in which cardiovascular irAE develops depending on the underlying cancer type. Furthermore, pre-existing CVD is indicated in the majority of patients with cardiovascular irAEs, specifically acute coronary syndrome (ACS) and heart failure. Created with Biorender

ICI immunotherapy is a promising and potent anti-cancer treatment but is associated with fatal cardiac irAEs, including myocarditis, pericarditis, and vasculitis. To date, only a few clinical risk factors have been associated with the development of these cardiac irAEs (Fig. 3). This review provides an overview of the current literature and data, which is summarized in Table 1, with the aim of highlighting these risk factors. The predominant risk factor for myocarditis is ICIs in combination with ICIs or TKIs. For pericarditis and vasculitis, the current literature does not observe this; however, more human data is needed. Tumor-specific factors also play a role in the development of irAEs as different profiles have been reported with ICI treatment. Patients with lung cancer seem to be more prone to develop ICI-mediated pericarditis, whereas myocarditis and vasculitis were more prevalent among melanoma patients. Females are also more likely to develop ICI-mediated myocarditis and pericarditis, which could possibly be linked to their predisposition to autoimmune disease, and pre-existing CVD, namely ACS and heart failure, was observed in the majority of patients with cardiovascular irAEs. In the future, large prospective cohort studies are warranted to further investigate and strengthen these abovementioned associations. Additionally, other risk factors should be explored, such as the microbiome as it seems to influence ICI-mediated irAEs, including cardiac irAEs [67]. A considerable problem with the ICI to date is that patients can become resistant, predominantly when the PD-1/PD-L1 axis is targeted. Promising ICIs are currently under investigation to overcome these issues.

**Table 1:** A current overview of potential risk factors for ICI-mediated myocarditis, pericarditis and vasculitis

Risk factor	ICI-mediated disease	Results	Literature
Treatment regimen			
ICI monotherapy vs Combination therapy	Myocarditis	- ROR of 5.62 [2.46–12.88] for anti-PD-1/PD-L1 vs anti-CTLA-4 monotherapy - ROR of 4.31 [2.86–6.38] for ICI combination vs monotherapy	[5]
		- 4.1-fold more prevalence of anti-PD-1/PD-L1 versus anti-CTLA-4	[13•]
		- 2.8-fold increased occurrence in anti-PD-1/LAG-3 vs anti-PD-1	[17••]
		- 3.4-fold increased occurrence in ICI combination vs monotherapy	[32]
	Pericardial disease	- ROR of 2.28 (1.27–4.12 95% CI) for PD-L1 or PD-1 treatment compared to CTLA-4	[5]
		- 6.7-fold more prevalence of anti-PD-1/PD-L1 versus anti-CTLA-4	[13•]
	Vasculitis	- Associated with ICI treatment; ROR 1.56 [1.25–1.94]	[5]
ICI and radiotherapy	Myocarditis	- Increased CD8 + lymphocytic cell filtrates in the heart, decreased cardiac output and fibrosis	[29]
		- Increased infiltrates of T-lymphocytes in the heart and lungs and increased levels of IFNγ	[30]
ICI and chemotherapy	Cardiovascular irAEs	- Significant increase in incidence of cardiovascular irAEs with a pooled risk ratio of 1.68 [1.07–2.64, *p* = 0.026]	[32]
	Myocarditis	- Incidence rate of 3.7% [1.62–9.1] for concomitant ICI and chemotherapy compared to 2.5% [0.77–5.85] for chemotherapy (1.48-fold increase)	[13•]
ICI and TKI	Myocarditis	- ROR of 36.9 [11.8–115.9] for pembrolizumab and axitinib - ROR of 55.6 [13.4–22.3] for avelumab and axitinib	[34]
Tumor type			
High tumor mutation burden	Any irAEs	- Malignancies with high TMB are associated with higher risk on developing any irAEs	[44]
Lung cancer	Cardiovascular irAEs	- More frequent in patients with NSCLC compared to small cell lung cancer (11.4% vs 6.7%)	[57]
		- Increased risk in patients with NSCLC compared to small cell lung cancer (42.3% vs 34.7%) with 2.1% developing myocarditis and 1.2% pericarditis within 1-year post-ICI initiation	[39]
	Pericarditis	- Significant increased the risk with HR of 5.46 [CI 2.96 to 10.10, *p* < 0.001]	[18]
Melanoma	Myocarditis and vasculitis	- More frequent in melanoma patients than in lung cancer patients; 41% for myocarditis and 60% for melanoma	[5]
Pre-existing CVD			
CVD	Cardiovascular irAEs	- Substantial fraction, 40–66%, with prior CVD	[13•, 54, 55]
	Myocarditis	- Increased risk associated with history of ACS and heart failure (HR 4.06 [1.15–14.3, *p* = 0.03] and 5.2 [1.4–18.7, *p* = 0.01], respectively)	[41]
Diabetes mellitus	Myocarditis	- More frequent in patients with ICI treatment than in controls, 34% vs 13%, *p* = 0.01, respectively - DM as an independent risk factor (OR = 1.96)	[58]
Sex-specific			
Sex	Cardiovascular irAEs	- Incidence rate ratio 3.340 [1.421–7.849, *p* = 0.006] for females	[40]
	Myocarditis	- Increased mortality (± 50%) among female *Ctla*^+*/−*^*Pdcd1*^*−/−*^ mice as compared to male ones	[49]
		- Female sex (OR, 1.92; 95% CI, 1.24–2.97; *p* = 0.004) significantly associated	[61]

### Future Perspective

The incidence of cardiovascular toxicities is expected to increase with the growing number of ICI being used in the clinics. Currently, the exact pathophysiological mechanisms behind the three cardiovascular irAEs covered in this review remain to be further elucidated. This knowledge will give new insights into cardiovascular immune homeostasis but might also be transferable to the understanding of other cardiac diseases and the role ICs might play. For now, baseline cardiac assessments are recommended to be performed for every patient before initiating ICI treatment and frequent monitoring of patients who may be at risk is advised to provide them with the best care.
